# Glutamine Is Required for M1-like Polarization of Macrophages in Response to Mycobacterium tuberculosis Infection

**DOI:** 10.1128/mbio.01274-22

**Published:** 2022-06-28

**Authors:** Qingkui Jiang, Yunping Qiu, Irwin J. Kurland, Karl Drlica, Selvakumar Subbian, Sanjay Tyagi, Lanbo Shi

**Affiliations:** a Public Health Research Institute, New Jersey Medical School, Rutgers Biomedical and Health Sciences, Rutgers, The State University of New Jersey, Newark, New Jersey, USA; b Stable Isotope & Metabolomics Core Facility, The Einstein-Mount Sinai Diabetes Research Center (ES-DRC), Albert Einstein College of Medicine, Bronx, New York, USA; c Public Health Research Institute and Department of Microbiology, Biochemistry, and Molecular Genetics, New Jersey Medical School, Rutgers Biomedical and Health Sciences, Rutgers, The State University of New Jersey, Newark, New Jersey, USA; Washington University School of Medicine in St. Louis

**Keywords:** M1-like polarization, immunometabolism, isotope tracing metabolomics, glutaminolysis, TCA cycle, *Mycobacterium tuberculosis*

## Abstract

In response to Mycobacterium tuberculosis infection, macrophages mount proinflammatory and antimicrobial responses similar to those observed in M1 macrophages activated by lipopolysaccharide (LPS) and interferon gamma (IFN-γ). A metabolic reprogramming to hypoxia-inducible-factor 1 (HIF-1)-mediated uptake of glucose and its metabolism by glycolysis is required for M1-like polarization, but little is known about other metabolic programs driving the M1-like polarization during infection. We report that glutamine serves as a carbon and nitrogen source for the metabolic reprogramming to M1-like macrophages. Widely targeted metabolite screening identified an association of glutamine and/or glutamate with highly affected metabolic pathways of M1-like macrophages. Moreover, stable isotope-assisted metabolomics of U^13^C glutamine and U^13^C glucose revealed that glutamine, rather than glucose, is catabolized in both the oxidative and reductive tricarboxylic acid (TCA) cycles of M1-like macrophages, thereby generating signaling molecules that include succinate, biosynthetic precursors such as aspartate, and itaconate. U^15^N glutamine-tracing metabolomics further revealed participation of glutamine nitrogen in synthesis of intermediates of purine and pyrimidine metabolism plus amino acids, including aspartate. These findings were corroborated by diminished M1 polarization from chemical inhibition of glutaminase (GLS), the key enzyme in the glutaminolysis pathway, and by genetic deletion of *GLS* in infected macrophages. Thus, the catabolism of glutamine is an integral component of metabolic reprogramming in activating macrophages and it coordinates with elevated cytosolic glycolysis to satisfy the cellular demand for bioenergetic and biosynthetic precursors of M1-like macrophages. Knowledge of these new immunometabolic features of M1-like macrophages should advance the development of host-directed therapies for tuberculosis.

## INTRODUCTION

As professional phagocytes, macrophages play essential roles in regulating tissue homeostasis and immune response to pathogens. Although traditionally classified into proinflammatory M1 and anti-inflammatory M2 states by their response to treatment with interferon gamma (IFN-γ) and lipopolysaccharide (LPS), as well as with interleukin-4 (IL-4) and IL-13 (1), the states of macrophage activation are often within the spectrum of M1 to M2 depending on microenvironmental factors and signaling molecules (2, 3). Recent advances in immunometabolism reveal that the polarization states of macrophages are closely associated with distinctive metabolic states. For example, M1 polarization is marked by increased expression of glucose-uptake transporters and isoenzymes of the glycolysis pathway, which leads to elevated glycolytic flux with increased lactate formation and secretion (4), similar to the Warburg effect (aerobic glycolysis) seen in cancer cells. In contrast, M2 polarization depends predominantly on mitochondrial oxidative metabolism (4, 5).

Amino acids also play important roles in macrophage activation (6). For example, it is well known that increased uptake of arginine and its catabolism by inducible nitric oxide synthase 2 (NOS2) produces NO, which is indispensable for the defense of M1 macrophages from invading pathogens. In contrast, arginase 1-mediated metabolism supports M2 polarization, tissue homeostasis, and repair (1). Additionally, catabolism of tryptophan by amino acid oxidases, including interleukin 4 Induced 1 and/or indoleamine 2,3-dioxygenase 1, with the formation of bioactive metabolites, such as kynurenine and kynurenic acid, promotes the generation of repressor macrophages and inhibition of Th1 immunity (7–10). Glutamine, which is consumed by immune cells at a rate similar to or higher than that of glucose (11), is an essential nutrient for effector functions of activated immune cells (12). However, a clear role for glutamine in macrophage polarization has not been established due to conflicting reports. For example, glutamine is proposed to be important for signaling by hypoxia-inducible factor 1 (HIF-1) and for mTOR1C activation in LPS-induced M1 macrophages (13, 14). In contrast, other studies argue that glutamine metabolism restricts the proinflammatory M1 state and favors M2 polarization (15–17). The latter idea derives from, at least in part, the glutamine-derived alpha ketoglutarate (α-KG) that enhances mitochondrial oxidative metabolism and inhibits HIF-1α by promoting the activity of HIF prolyl hydroxylases (15). Thus, the role of glutamine during infection with Mycobacterium tuberculosis remains uncertain.

Macrophage infection by various bacteria, including M. tuberculosis, the etiological agent of tuberculosis (TB), leads to an initial robust proinflammatory response that resembles that seen with classically activated M1 macrophages (18, 19). Previous studies, including our own, reveal a metabolic reprogramming involving HIF-1 induction and increased glycolytic flux, indicating a role for glycolysis in the proinflammatory and antimicrobial responses of M. tuberculosis-infected murine bone marrow-derived macrophages (BMDMs) and mouse lungs (20–25). A similar metabolic reprogramming is also required to activate human alveolar and monocyte-derived macrophages in response to M. tuberculosis infection, as inhibition of glycolysis by 2-deoxyglucose dampens the M1-like polarization and promotes survival of M. tuberculosis (26). Reports also show that macrophages from ontologically distinct lineages exhibit various degrees of immunometabolic features in response to M. tuberculosis infection (25, 27). For example, alveolar macrophages display a glycolytic state and proinflammatory response less pronounced than those of BMDMs or interstitial macrophages originating from bone marrow-derived monocytes, thereby favoring M. tuberculosis survival and growth (25, 28). To date, the other metabolic programs propelling M1-like polarization during M. tuberculosis infection have not been clearly defined. Given that M. tuberculosis can modulate the immunometabolic response of infected murine and human macrophages to survive and persist (29, 30), a better understanding of metabolic programs of host cells will help develop host-directed therapies to promote bacterial clearance.

In the present work, we dissected the immunometabolic features of M. tuberculosis infection-induced M1-like macrophages using (i) single-molecule RNA fluorescence *in situ* hybridization (sm-RNA-FISH), (ii) metabolic profiling and stable isotope-assisted metabolomics, and (iii) pharmacological and genetic manipulations targeting glutaminolysis. We report that, apart from increased glucose catabolism by glycolysis, glutamine and its direct metabolite, glutamate, serve as carbon and nitrogen sources for the synthesis of biosynthetic precursors involved in multiple metabolic pathways of the activating macrophages. Thus, glutamine is central to the proinflammatory and antimicrobial responses of M1-like polarization against M. tuberculosis.

## RESULTS

### Characterization of infected BMDMs by sm-RNA-FISH.

**FIG 1:**
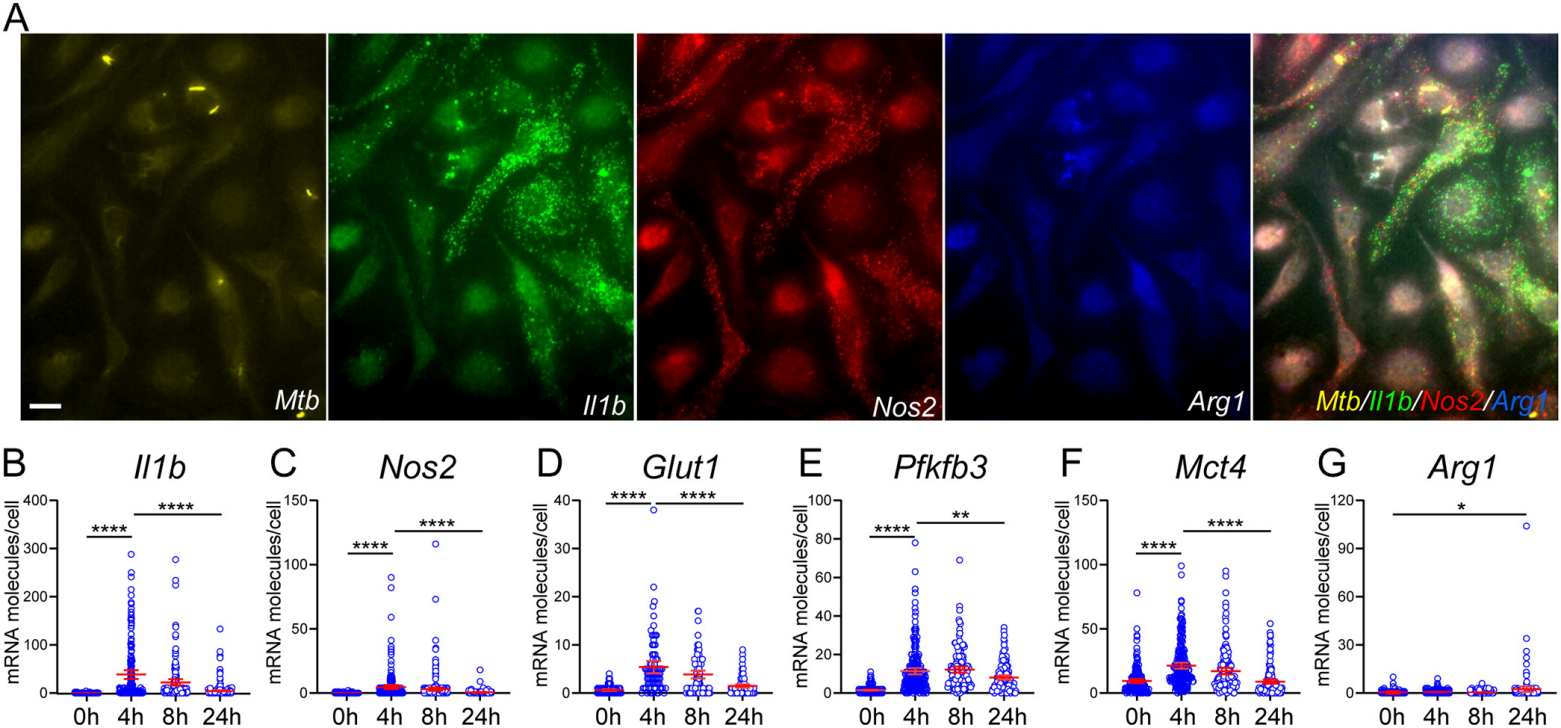
Single-cell mRNA analysis of immunometabolic markers in M. tuberculosis-infected BMDMs. BMDMs seeded on coverslips were probed in 3-plex hybridization reactions using sm-RNA-FISH probes labeled with transcript-specific probe sets (about 50 oligonucleotides for each mRNA) that were coupled to tetramethylrhodamine (TMR), Texas Red, or Cy5 fluorophores. Images were acquired in Z-stacks of different fields of cells in different channels. Fluorescence spots corresponding to single mRNA molecules in individual cells were counted in the merged Z-stacks using a custom image-processing algorithm implemented in MATLAB. (A) Representative images of GFP-labeled M. tuberculosis (*Mtb*; yellow) and mRNA molecule spots for *Il1b* (green), *Nos2* (red), and *Arg1* (blue) at 8 hpi in Z-stacks of the same field of cells in different channels and merged together. (B to G) Changes of respective mRNA molecules of immunometabolic markers in individual cells at 0, 4, 8, and 24 hpi. A total of ~100 to 150 cells were analyzed by sm-RNA-FISH at the indicated times p.i. Each circle represents one cell. Representative data are shown as means ± 95% CI (confidence interval) from at least three independent experiments. Statistical significance at *, *P < *0.05, **, *P < *0.01, ***, *P < *0.001 and ****, *P < *0.0001 was based on two-tailed student’s *t* test. The scale bar in panel A is 10 μm.

Activation of murine BMDMs during M. tuberculosis infection is accompanied by an early M1-like response and a metabolic remodeling to glycolysis for at least 8 to 12 h postinfection (hpi) (23, 31). That is followed by a late resolution/adaptation phase associated with dampening of M1 polarization and the recovery of mitochondrial oxidative metabolism at 24 hpi and beyond (23, 31). Such a shift in immunometabolic states of infected macrophages corresponds to changes in growth dynamics and physiology of M. tuberculosis inside the host cells (32). We used sm-RNA-FISH, which provides specific detection and quantification of single molecules of target mRNAs *in situ* at the single-cell level (33), to characterize the expression of several immunometabolic markers of infected murine BMDMs. Consistent with the transcriptomic dynamics of M. tuberculosis-infected murine BMDMs (23, 31), infected BMDMs at 4 to 8 hpi showed increased numbers of mRNA molecules of the M1 markers IL-1β and NOS2, as well as the Warburg effect enzymes, GLUT1 (glucose transporter 1), PFKFB3 (6-phosphofructo-2-kinase/fructose-2,6-bisphosphatase 3, a key regulatory enzyme for glycolysis), and MCT4 (the major lactate efflux transporter) (Fig. 1A to F). These increases were followed by a decrease at 24 hpi. In contrast, mRNA molecules for ARG1, an M2 marker, showed a clear induction only at 24 hpi (Fig. 1G).

We observed considerable heterogeneity among the cells, with only some cells expressing a significant number of mRNA molecules for a given marker (Fig. 1A). For example, cells that expressed a significant number of *Il1b* mRNA molecules did not express significant *Nos2* RNAs and vice versa. Indeed, a slight negative correlation was seen between the two mRNA molecule species in single cells (see Fig. S1 in the supplemental material). This result is consistent with NO inhibiting IL-1β at the transcription level (21) and with high cell-to-cell heterogeneity for the expression of cytokines in single macrophages (34).

10.1128/mbio.01274-22.1 FIG S1 Negative correlation between mRNA copy numbers of NOS2 and those of IL-1β in M. tuberculosis-induced M1-like macrophages. Individual cells that express mRNA molecules for both NOS2 and IL-1β in M. tuberculosis-infected BMDMs at 8 hpi from Fig. 1A were plotted. Each circle represents one cell, and its location on the graph identifies the number of molecules of the mRNA species indicated on the two axes. Cells that express a significant number of marker mRNAs (red circles) above a threshold (defined as 1/10 of maximum level for each species) were segregated from those that express no or few molecules (green circles). Observation of a slight negative correlation between mRNA molecules of NOS2 and IL-1β indicates that cells that express a significant amount of one marker express no or few of the other marker. Data are derived from Fig. 1A. Correlation was performed using the correlation coefficient (corrcoef) function in MATLAB. Download FIG S1, TIF file, 0.8 MB. Copyright © 2022 Jiang et al. 2022 Jiang et al. https://creativecommons.org/licenses/by/4.0/ This content is distributed under the terms of the Creative Commons Attribution 4.0 International license.

The mRNA expression dynamics of the immunometabolic markers in infected BMDMs also correlated with the production of IL-1β, as measured in the culture supernatant (Fig. S2). Thus, our data from murine BMDM infection are in line with the switch of host cellular metabolism toward aerobic glycolysis in human peripheral blood mononuclear cells (PBMCs) obtained from patients with active TB, in M. tuberculosis-stimulated PBMCs, and in infected human primary macrophages (26, 35).

10.1128/mbio.01274-22.2 FIG S2 IL-1β secretion from M. tuberculosis-infected murine macrophages. Murine BMDMs were infected, and culture supernatants were collected at 0, 4, 8, and 24 hpi. IL-1β protein in cell supernatant was measured by the mouse IL-1β uncoated ELISA kit, and the kinetics of IL-1β secretion at the indicated time intervals was calculated. Representative data are shown from three independent experiments; statistical significance at *, *P < *0.05, **, *P < *0.01, ***, *P < *0.001, and ****, *P < *0.0001 was based on two-tailed student’s *t* test. Download FIG S2, TIF file, 0.4 MB. Copyright © 2022 Jiang et al. 2022 Jiang et al. https://creativecommons.org/licenses/by/4.0/ This content is distributed under the terms of the Creative Commons Attribution 4.0 International license.

### Metabolite screening identifies highly impacted metabolic pathways associated with glutamine.

**FIG 2:**
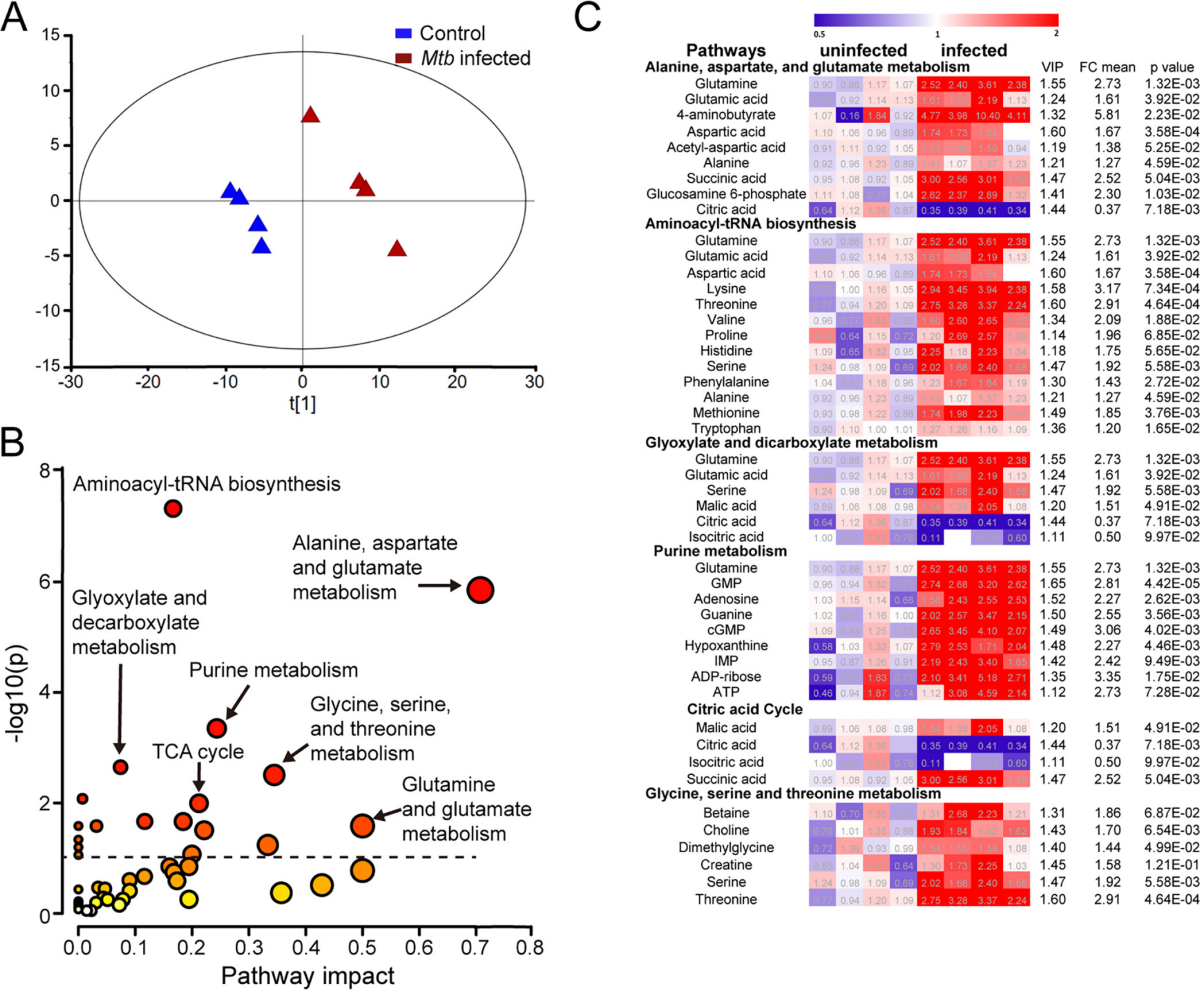
Identification of highly impacted metabolic pathways during M. tuberculosis-induced M1-like polarization by widely targeted small-metabolite screening. Metabolites extracted from M. tuberculosis-infected BMDMs at 8 hpi and uninfected controls were analyzed by the QTRAP 6500+ LC-MS/MS systems. (A) Separation of M1-like macrophages from uninfected controls as scores plot from PLS-DA modeling. (B) Highly impacted metabolic pathways in M1-like macrophages. The differential metabolites with variable importance in the projection (VIP) score of >1 from the PLS-DA modeling of the two groups were subjected to pathway enrichment analysis by the Metaboanalyst (V5.0). The circle size denotes the altitude of pathway impact, and color darkness represents the extent of significance. (C) Heatmaps of differential metabolites with VIP of >1 in highly impacted metabolic pathways. Data are shown as normalized values to the corresponding mean value of the uninfected group (4 biological repeats per group).

To better understand the metabolic programs driving M1-like polarization, we carried out a widely targeted screen of small metabolites using the QTRAP 6500+ liquid chromatography-tandem mass spectrometry (LC-MS/MS) systems (ABSciex) in M. tuberculosis-infected BMDMs at 8 hpi. A total of 169 metabolites were detected having a coefficient of variation (CV) cutoff of 30%, which is commonly used and/or recommended for metabolomics data in the literature (36–38). Multivariate analysis of the data sets was performed using SIMCA-p software; the partial least-squares-discriminant analysis (PLS-DA) model revealed distinct differences in the metabolic profiles between infected macrophages and uninfected controls (Fig. 2A). Key metabolites that contributed to this distinction were identified by using the variable importance in the projection (VIP) score of >1.

In the infected BMDMs, we observed increased levels of intermediates, such as fructose 1,6-bisphosphate and 3-phosphoglyceric acid of glycolysis, as well as pentose phosphate of the pentose phosphate pathway (PPP) (Fig. S3). These findings are consistent with metabolic reprogramming of glucose metabolism during M. tuberculosis-induced macrophage activation (20, 23, 26). Using pathway enrichment analysis (Metaboanalyst [V5.0]) for the differential metabolites having a VIP score of >1, we identified highly impacted metabolic pathways associated with macrophage activation toward the M1-like phenotype (Fig. 2B). A detailed examination of metabolites among the impacted pathways revealed that glutamine and its direct metabolite, glutamate, had positive associations with multiple pathways of macrophage activation, including purine metabolism, glyoxylate dicarboxylate metabolism, the tricarboxylic acid (TCA) cycle, and alanine, aspartate, and glutamate metabolism. The latter pathway was the most affected, having a low −log_10_(p) of 5.9 and a high pathway impact factor of 0.71 (false-discover rate [FDR], 5.8859E−5) (Fig. 2B and C). We verified these observations using a more stringent CV cutoff of ≤20%, which revealed similarly impacted pathways that included alanine, aspartate, and glutamate metabolism, as well as glutamine and glutamate metabolism being the two most affected (Fig. S4).

10.1128/mbio.01274-22.3 FIG S3 Heatmaps of additional differential metabolites with variable importance in the projection (VIP) score of >1 in M1-like macrophages. Metabolites from infected murine BMDMs at 8 hpi and uninfected control cells were subjected to identification and quantification by the QTRAP 6500+ LC-MS/MS systems (ABSciex). Shown are heatmaps of differential metabolites with a VIP of >1 from PLS-DA modeling of two groups, which were not listed in Fig. 2C. Data are shown as normalized values to the corresponding mean value of the uninfected group (4 biological repeats per group). Download FIG S3, TIF file, 2.6 MB. Copyright © 2022 Jiang et al. 2022 Jiang et al. https://creativecommons.org/licenses/by/4.0/ This content is distributed under the terms of the Creative Commons Attribution 4.0 International license.

10.1128/mbio.01274-22.4 FIG S4 Identification of highly impacted metabolic pathways in M1-like macrophages by widely targeted small-metabolite screening with a CV cutoff of 20%. Metabolite extraction and analysis by the QTRAP 6500+ LC-MS/MS systems from M. tuberculosis-infected BMDMs at 8 hpi and uninfected controls (4 biological repeats per group) were described in Fig. 2. The differential metabolites with VIP score of >1 from the PLS-DA modeling of the two groups with a CV cutoff of 20% were subjected to pathway enrichment analysis by the Metaboanalyst (V5.0). Alanine, aspartate, and glutamate metabolism, as well as glutamine and glutamate metabolism, are the two most impacted pathways. The circle size denotes the altitude of pathway impact, and color darkness represents the extent of significance. Download FIG S4, TIF file, 1.9 MB. Copyright © 2022 Jiang et al. 2022 Jiang et al. https://creativecommons.org/licenses/by/4.0/ This content is distributed under the terms of the Creative Commons Attribution 4.0 International license.

We also compared the metabolite changes in infected BMDMs between 8 and 24 hpi. These comparisons showed increased accumulation for some metabolites in the impacted pathways by 24 hpi, albeit at a smaller amount than at the first 8 hpi; some metabolites showed decreased levels (Fig. S5). These observations are consistent with the decelerated bioenergetics observed in M. tuberculosis-infected murine and human macrophages (29, 30). Overall, the change of metabolite profiles in M. tuberculosis-infected BMDMs correlated with mRNA dynamics of infected macrophages transitioning from the M1-like polarization state to the adaptation/resolution phase of infection, as characterized by transcriptomic profiling (23) and by sm-RNA-FISH in Fig. 1.

10.1128/mbio.01274-22.5 FIG S5 Change of metabolic state of M. tuberculosis-infected BMDMs between 8 and 24 hpi. Heatmaps of differential metabolites in Fig. 2C and Fig. S3 at 8 and 24 hpi. Metabolites extracted from M. tuberculosis-infected BMDMs at 8 and 24 hpi were analyzed by the QTRAP 6500+ LC-MS/MS systems. Data are shown as normalized values to the corresponding mean value of infected group at 8 hpi (4 biological repeats per group). FC, fold change. Download FIG S5, TIF file, 2.3 MB. Copyright © 2022 Jiang et al. 2022 Jiang et al. https://creativecommons.org/licenses/by/4.0/ This content is distributed under the terms of the Creative Commons Attribution 4.0 International license.

Strong evidence for glutamine involvement in M1-like polarization is the increased accumulation in infected BMDMs of 4-aminobutyrate (GABA), the central metabolite of the GABA shunt that supplies glutamate-derived succinate to the TCA cycle. The GABA shunt is associated with M1 polarization, as inhibition of the shunt by vigabatrin (an irreversible inhibitor of GABA transaminase) decreases succinate concentration, leading to reduction of HIF-1 and IL-1β in LPS-activated M1 macrophages (14). The identification of increased 2-hydroxyglutaric acid (2HG) (Fig. S3), which is converted from glutamine-derived α-KG in cancer cells having deficient activity of isocitrate dehydrogenase (IDH) 1 or 2 due to mutation and in mammalian cells under hypoxia (39–43), also suggests a connection of glutamine catabolism to the TCA cycle during M1-like polarization.

Given that glutamine and/or glutamate participate in the synthesis of glutathione (GSH; the reduced form), a major intracellular, small-molecule antioxidant in proinflammatory immune cells (44, 45), increased levels of glutamine, glutamate, GSH, and oxidized glutathione (GSSG) (Fig. S3), as determined by widely targeted small-metabolite screening, suggest a role for glutamine metabolism in maintaining redox homeostasis in M1-like macrophages. This idea is supported by upregulation in M. tuberculosis-infected BMDMs of *Gclc* and *Gclm*, which encode, respectively, the catalytic and modifier subunit of glutamate-cysteine ligase (23), the first rate-limiting enzyme in GSH synthesis (46). Moreover, glutamine is an essential precursor for *de novo* GSH biosynthesis during murine T cell differentiation and proliferation (44).

Elevated GSH synthesis is also associated with increased antiporter system xC^−^, which consists of xCT (also known as SLC7A11) and its chaperone CD98 (SLC3A2) (47). xCT plays an important role in maintaining intracellular GSH levels and redox balance by mediating the uptake of cystine, another precursor for GSH synthesis (48). Increased xCT expression in macrophages correlates with susceptibility of host cells to M. tuberculosis infection by regulating antimicrobial function and inflammation (49). As expected, we observed a similar induction of *xCT* mRNA in M. tuberculosis-infected M1-like macrophages (Fig. S6A). Collectively, the findings from metabolite screening of infected macrophages suggest an important role for glutamine in M. tuberculosis-induced M-1 like polarization.

10.1128/mbio.01274-22.6 FIG S6 Increased expression of *xCT* in infected BMDMs and decreased GSH/GSSG in infected THP1 *Gls* KO macrophages. BMDMs were infected with M. tuberculosis and subjected to *xCT* mRNA analysis by sm-RNA-FISH as described in Fig. 1. A total of ~100 to 150 cells were analyzed by sm-RNA-FISH at the indicated times p.i. Each circle in panel A represents one cell. Representative data are shown as means ±95% CI (confidence interval) from at least three independent experiments. THP1 *Gls* KO macrophages and WT control cells were seeded into 96-well plates and infected by M. tuberculosis. Intracellular GSH and GSSG were quantified using the Promega GSH/GSSG-Glo assay kit by measuring luminescence intensity in Agilent Cytation 5. Representative data in panel B are shown as means ± S.D. from 4 biological replicates. Statistical significance at *, *P < *0.05, **, *P < *0.01, ***, *P < *0.001, and ****, *P < *0.0001 was based on two-tailed student’s *t* test. Download FIG S6, TIF file, 0.5 MB. Copyright © 2022 Jiang et al. 2022 Jiang et al. https://creativecommons.org/licenses/by/4.0/ This content is distributed under the terms of the Creative Commons Attribution 4.0 International license.

### Tracing metabolomics of U^13^C glutamine and glucose identifies anaplerosis of glutamine carbons through both the oxidative and reductive TCA cycle.

**FIG 3:**
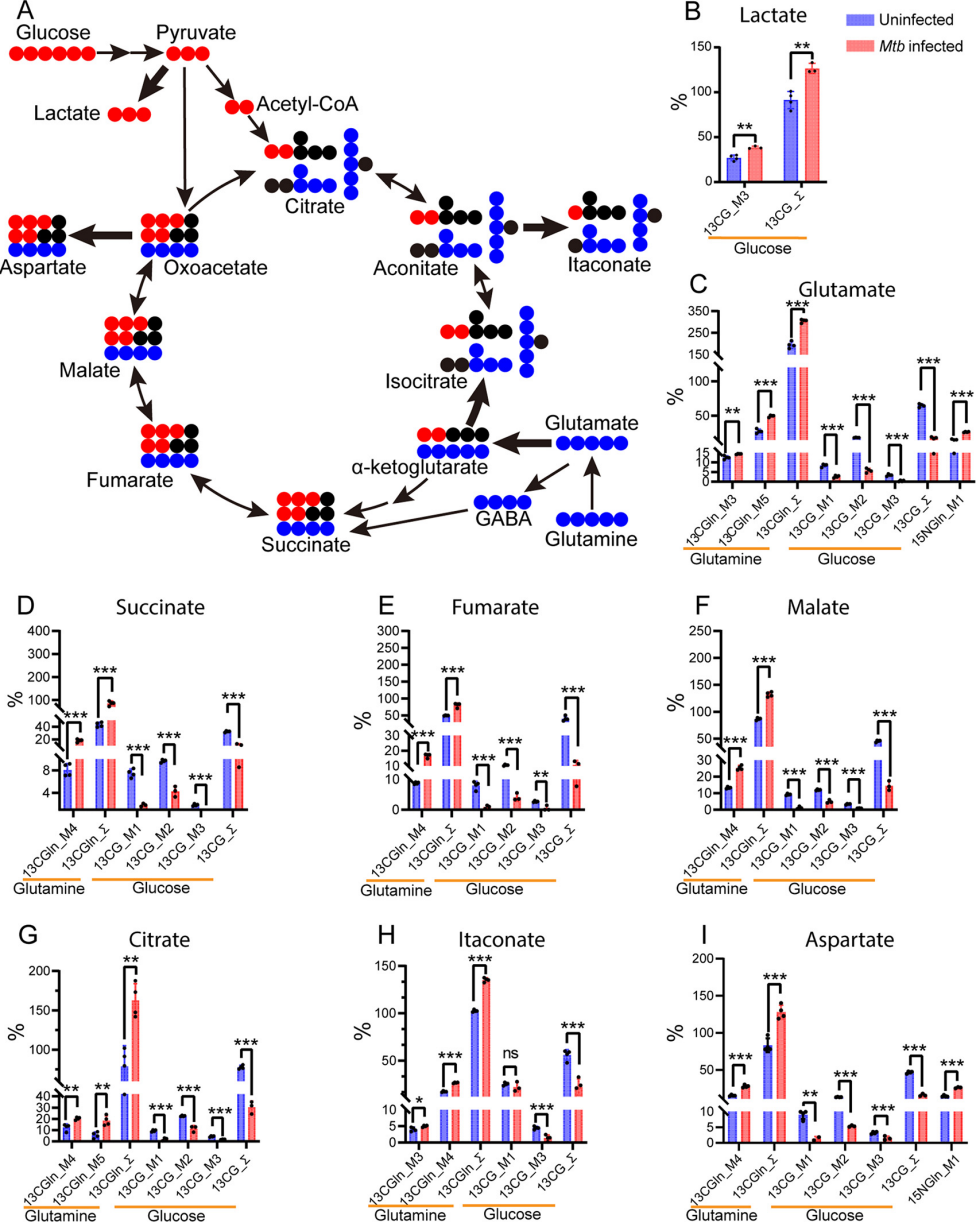
Isotope labeling distribution pattern of U^13^C glucose and glutamine and U^15^N glutamine during the M1-like polarization. (A) Diagram of ^13^C distribution from the catabolism of U^13^C glucose and U^13^C glutamine in glycolysis and/or the TCA cycle. Catabolism of U^13^C glucose generates M + 3 glycolytic intermediates and M + 2 (via pyruvate oxidation) and M + 3 (via pyruvate carboxylation) TCA cycle intermediates/derivatives. M + 5 glutamate, the direct metabolite of U^13^C glutamine, can enter the TCA cycle in the form of alpha ketoglutarate (α-KG) and/or via the GABA shunt. Increased ^13^C distribution from U^13^C glucose catabolism in glycolysis (B) with the generation of M + 3 lactate and (C to I) from the catabolism of U^13^C glutamine in the oxidative and reductive TCA cycle resulting in the generation of TCA cycle intermediates/derivatives. M1-like polarization was marked by diverting the ^13^C glucose carbon distribution from the TCA cycle M + 2, M + 1, and M + 3 intermediates to the formation of M + 3 lactate (A). In contrast, catabolism of U^13^C glutamine led to increased ^13^C distribution in the form of M + 4 TCA cycle intermediates/derivatives, including succinate (D), fumarate (E), malate (F), itaconate (H), and aspartate (I), as well as M + 4 and M + 5 citrate (G), indicating the simultaneous operation of both the oxidative and reductive TCA cycle. Increased ^15^N distribution from U^15^N glutamine to M + 1 aspartate (I) also indicates glutamine being a nitrogen source for the formation of nonessential amino acid aspartate. Solid red and blue circles represent ^13^C from U^13^C glucose and U^13^C glutamine, respectively. Thick arrows indicate increased ^13^C carbon flux into the formation of lactate and the TCA cycle intermediates or derivatives, respectively. Enrichment calculated from U^13^C glucose was marked as 13CG_M1, 13CG_M2, 13CG_M3, and 13CG_Σ. Σ was calculated with M1 × 1 + M2 × 2…+ Mn × *n*. Enrichment calculated from U^13^C glutamine was marked as 13CGln_M3, 13CGln_M4, 13CGln_M5, and 13CGln_Σ. Enrichment calculated from ^15^N glutamine was marked as 15NGln_M1. Data are shown as means ± standard deviation (S.D.) from 3 to 4 biological replicates. Statistical significance at *, *P < *0.05, **, *P < *0.01, ***, *P < *0.001 and ****, *P < *0.0001 was based on two-tailed student’s *t* test.

To determine the contribution of glutamine as a carbon source for the metabolic program of macrophage activation, we used U^13^C glutamine to track its metabolic signature in M. tuberculosis-infected M1-like macrophages by gas chromatography/time-of-flight mass spectrometry (GC-TOF/MS) (50). Infected BMDMs were cultured for 8 h in Dulbecco’s modified Eagle’s medium (DMEM) supplemented with 4 mM 50% U^13^C glutamine; then, cellular metabolites were extracted, derivatized with silylation reagents, and analyzed for isotope enrichment (50). Carbons of glutamine can enter the TCA cycle in the form of glutamate-derived α-KG and/or as succinate from the GABA shunt (Fig. 3A). The labeling pattern of U^13^C glutamine carbons from the first turn of the oxidative TCA cycle would generate four ^13^C-labeled intermediates, such as succinate, fumarate, malate, and citrate (detected as M + 4 by mass spectroscopy). The labeling pattern of U^13^C glutamine from the first turn of the reductive TCA cycle would produce the M + 5 intermediates citrate and isocitrate (Fig. 3A). As shown in Fig. 3D to I and Table S1, increased enrichment of ^13^C isotopes was predominantly for M + 4 intermediates (~1.5- to 2.2-fold) that included succinate, fumarate, malate, itaconate, and citrate, as well as for M + 5 citrate (~3.3-fold) in infected BMDMs relative to uninfected controls. Thus, increased glutamine carbon flux to TCA cycle intermediates arises from both the oxidative and reductive directions. This conclusion is supported by the simultaneous increase in enrichment for both M + 4 (~1.6-fold) and M + 5 citrate (~3.3-fold) in infected BMDMs (Table S1), with the latter, which derives from reductive carboxylation of glutamine-derived α-KG, being more pronounced (Fig. 3G).

10.1128/mbio.01274-22.9 TABLE S1 Source data of isotope tracing metabolomics with U^13^C glutamine, U^13^C glucose, and U^15^N glutamine. Download Table S1, XLSX file, 0.03 MB. Copyright © 2022 Jiang et al. 2022 Jiang et al. https://creativecommons.org/licenses/by/4.0/ This content is distributed under the terms of the Creative Commons Attribution 4.0 International license.

The decrease of citrate and isocitrate in infected BMDMs (Fig. 2C) and increased enrichment of M + 4 and M + 5 citrate from U^13^C glutamine in both the oxidative and the reductive directions of the TCA cycle suggest an outflow of mitochondrial citrate. Indeed, the ~10-fold increase in the ratios between total itaconate and total citrate in infected BMDMs (Fig. S7) is consistent with high-level induction of the aconitate carboxylase-1 gene (*Acod1* or *Irg1*), whose product catalyzes the formation of itaconate in M1 macrophages (23, 31, 51, 52). This conclusion is supported by the increased enrichment of M + 4 itaconate (Fig. 3H), which could be derived from the oxidative TCA cycle-generated M + 4 and/or the reductive TCA cycle-generated M + 5 citrate. The enhanced formation of itaconate, and its inhibition of succinate dehydrogenase activity (35), may be a driving force in directing the flux of glutamine carbons toward succinate, as reported recently (53).

10.1128/mbio.01274-22.7 FIG S7 Increased formation of itaconate from citrate in infected M1-like macrophages. Total itaconate and citrate were derived from labeling levels of each metabolite in comparison to their M0 counterparts from the U^13^C glutamine tracing experiment and normalized to the mean values of uninfected controls. Shown are the ratios between total itaconate and total citrate. Data are shown as means ± S.D. from 3 to 4 biological replicates. Statistical significance at *, *P < *0.05, **, *P < *0.01, ***, *P < *0.001, and ****, *P < *0.0001 was based on two-tailed student’s *t* test. Download FIG S7, TIF file, 0.3 MB. Copyright © 2022 Jiang et al. 2022 Jiang et al. https://creativecommons.org/licenses/by/4.0/ This content is distributed under the terms of the Creative Commons Attribution 4.0 International license.

Importantly, we also observed very low enrichment of M + 3 TCA cycle intermediates, such as malate, fumarate, and succinate in M1-like macrophages (Table S1). These data indicate that the reductive TCA cycle-derived M + 5 citrate is not utilized for *de novo* fatty acid synthesis through the cytosolic citrate lyase-mediated pathway but rather that it is redirected to the formation of itaconate in M1-like macrophages. This finding contrasts with the utilization of M + 5 citrate for fatty acid synthesis that consequently leads to the generation of M + 3 TCA cycle intermediates in cancer cells under hypoxia and impaired respiration (54, 55).

When we examined the labeling distribution of glutamine carbons into other intermediates of central metabolism, including glycolysis and PPP, no significant ^13^C labeling was found in glycolysis intermediates such as lactate and pyruvate. These data indicate that glutamine is not a significant carbon contributor to glycolytic intermediates via gluconeogenesis. Importantly, we observed increased enrichment of ^13^C in M + 4 aspartate (~1.9-fold) in infected BMDMs (Fig. 3I and Table S1), which probably derives from transamination reactions of the TCA cycle intermediate oxaloacetate (OAA) (56). These data demonstrate that glutamine also serves as a major carbon donor for *de novo* synthesis of the nonessential amino acid aspartate during M1-like polarization. Taken together, the U^13^C glutamine tracing metabolomics data indicate that during M 1-like polarization, glutamine replenishes TCA cycle carbon flux for the synthesis of signaling molecules such as succinate, itaconate and nonessential amino acids such as aspartate.

To confirm that glutamine rather than glucose serves as a major carbon source for the TCA cycle during M1-like polarization, we analyzed the metabolic signature of U^13^C glucose by GC-TOF/MS (50). Infected BMDMs were cultured for 8 h in DMEM supplemented with 25 mM 50% U^13^C glucose, and cellular metabolites were extracted for isotope enrichment analysis. The labeling pattern of U^13^C glucose from glycolysis and the first turn of the TCA cycle would result in the generation of M + 3 glycolytic intermediates plus M + 2 and M + 3 TCA cycle intermediates from mitochondrial pyruvate dehydrogenase (PDH)- and pyruvate carboxylase (PC)-mediated pathways, respectively (Fig. 3A). As expected, the increased enrichment of ^13^C M + 3 lactate (~12%) in M. tuberculosis-infected BMDMs, relative to that in uninfected controls (Fig. 3B), agreed with increased glycolytic flux from glucose to lactate during the M1-like polarization (20, 26). In contrast to the labeling distribution of U^13^C glucose in uninfected controls, which was marked with ^13^C isotope distribution such as M + 2, M + 3, and M + 1 (the latter two can be deriving from the second turn of the mitochondrial PDH activity) into TCA intermediates and derivative glutamate (Fig. 3C to I), M1-like polarization during M. tuberculosis infection was marked by decreased enrichment of ^13^C isotopes for the TCA cycle intermediates, as well as their sum (Σ_m_^n^) (Fig. 3D to I and Table S1). Those intermediates with changed sum included succinate (~2.9-fold), fumarate (~3.5-fold), malate (~3.1-fold), and citrate (~2.6-fold), plus their derivatives itaconate (~2.1-fold), aspartate (~2.7-fold), and glutamate (~3.8-fold).

The results described above support our previous work that describes diminished PDH function associated with decreased carbon flux from glucose-derived pyruvate to the TCA cycle in M. tuberculosis-infected macrophages (23). In addition, given that itaconate is derived from M + 2 citrate precursor, our observation of little change in M + 1 itaconate (Fig. 3H), compared with a 2-fold decrease in M + 2 citrate (Fig. 3G) in infected M1-like macrophages, indicates a high rate of conversion of newly synthesized citrate toward the formation of itaconate in infected macrophages. This result is consistent with high-level induction of *Irg1* in infected macrophages (31, 52), despite the decrease in overall flux to itaconate from glucose, as discussed above. Taken together, these data demonstrate the rerouting of ^13^C glucose carbon flux from the TCA cycle to glycolysis during M1-like polarization. This rerouting is consistent with the presence of the Warburg effect in M. tuberculosis*-*infected macrophages (20, 23, 26) and with the observation that glucose is not a major carbon donor for the TCA cycle and aspartate synthesis in M1 macrophages (57).

### Tracing metabolomics of U^15^N glutamine identifies glutamine as a nitrogen source for the synthesis of nucleotides and amino acids.

By using U^15^N glutamine as a potential nitrogen source, we tracked the incorporation of glutamine nitrogen in the synthesis of nitrogen-containing compounds in M1-like macrophages by GC-TOF/MS (50). Infected BMDMs were cultured for 8 h in DMEM supplemented with 4 mM 50% U^15^N glutamine, and cellular metabolites were extracted for isotope enrichment analysis. Consistent with increased purine metabolism during M1-like polarization, as seen in the unlabeled metabolomics studies discussed above, we found increased enrichment of ^15^N glutamine in key metabolites of purine metabolism, including hypoxanthine (M + 1, ~4.7-fold; M + 2, ~2.5-fold), adenine (~1.5-fold for both M + 1 and M + 2), and uric acid (~2.5-fold for both M + 1 and M + 2) (Table S1). Since the generation of hypoxanthine and adenine is associated with nucleotide salvage pathways (57), the enrichment of glutamine nitrogen in their formation suggests a role for glutamine as a nitrogen source in the synthesis of nucleotides during M1-like polarization. In addition, enrichment of M + 1 and M + 2 uric acid is consistent with the finding that its formation via xanthine oxidase, arising from the catabolism of hypoxanthine and/or xanthine, is required for M1 polarization through xanthine oxidase-mediated reactive oxygen species production and signaling (58). Increased enrichment for M + 1 (~1.8-fold) and M + 2 (~2.7-fold) uracil, which can be recycled by uridine phosphorylase or uracil phosphoribosyltransferase, also indicates an involvement of glutamine in the pyrimidine metabolism of M1-like macrophages.

Glutamine nitrogen also participated in the synthesis of nonessential amino acids. M + 1 aspartate (Fig. 3I), glutamate (Fig. 3C), and alanine were ~1.7- to 2.0-fold higher in M1-like macrophages than in uninfected controls (Table S1). In particular, the finding that glutamine supplies both carbon and reduced nitrogen for the formation of aspartate suggests a potentially important role of aspartate in M1-like macrophages. Indeed, aspartate has been shown to promote IL-1β secretion in M1 macrophages by boosting the activation of HIF-1α (59). In regard to other amino acids, we detected only weak labeling of U^15^N glutamine into valine and isoleucine (Table S1). This result is consistent with tracing studies of various amino acids showing that activation of M. tuberculosis-infected human THP1 macrophages is associated with the direct uptake for most amnio acids from the culture medium rather than from the transamination of glutamine (60). Collectively, these data clearly demonstrate that glutamine serves as a nitrogen source for the synthesis of nucleotides and aspartate during M1-like polarization.

### Chemical inhibition and genetic manipulation of the glutaminolysis pathway diminish M1-like polarization.

**FIG 4:**
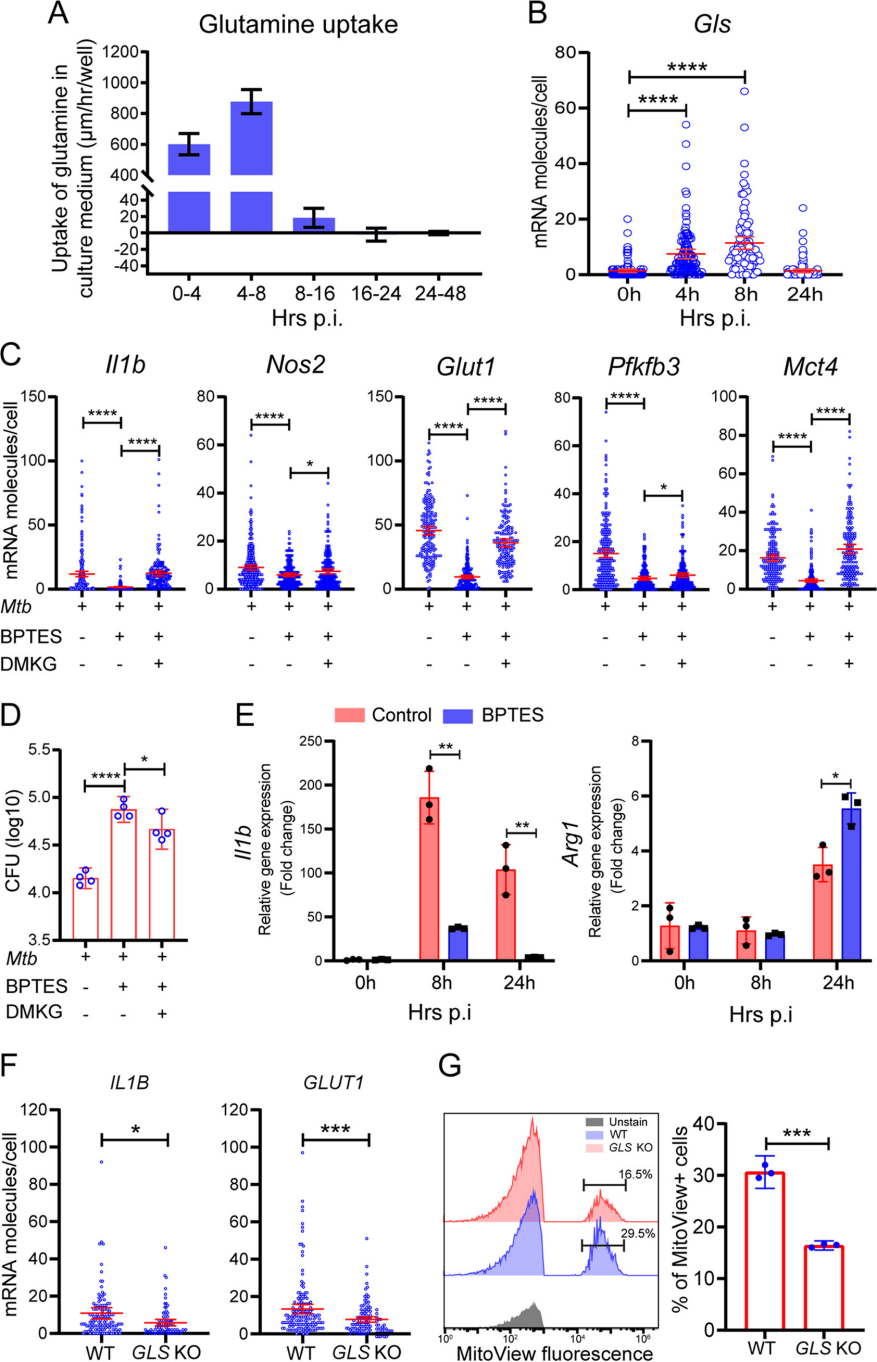
Requirement of glutamine for M1-like polarization. BMDMs were infected with M. tuberculosis, and cells and supernatants were collected at various times for single-cell mRNA analysis by sm-RNA-FISH and for measurement of glutamine uptake/utilization. Glutaminase (GLS) inhibitor BPTES was added to sets of cultures for GLS inhibition experiments at a final concentration of 10 μM. For rescue experiments, 1.5 mM dimethyl α-ketoglutarate (DMKG) was added to sets of cultures treated with the inhibitor. (A) High rate of glutamine uptake/utilization by M. tuberculosis-infected macrophages corresponding to the M1-like polarization. Cell culture supernatants collected at the indicated times were subjected to glutamine determination using the glutamine/glutamate-glo assay kit. Kinetics of glutamine uptake/utilization (μm per hour per well) were calculated based on its changes in the culture medium. Data are shown as means ± S.D. from three independent experiments. (B) Increased mRNA molecules for glutaminase gene *Gls* in M1-like macrophages. *Gls* mRNA molecules in infected BMDMs were detected and analyzed by sm-RNA-FISH as described in Fig. 1. (C) Diminished M1-like polarization by GLS inhibition with BPTES and alleviation of the inhibition by treatment with DMKG. mRNA molecules in infected BMDMs, with or without 10 μM BPTES and/or 1.5 mM DMKG at 8 hpi, were analyzed for *Il1b*, *Nos2*, *Glut1*, *Pfkfb3*, and *Mct4* by sm-RNA-FISH as described in Fig. 1. (D) Enhanced M. tuberculosis growth by GLS inhibition with BPTES. CFU of M. tuberculosis was determined by plating assay of cell lysates of infected BMDMs with indicated treatments at day 3 p.i. (E) Increased expression of *Arg1* and decreased *Il1b* in infected and BPTES-treated BMDMs. (F and G) Expression of *Il1b* and *Arg1* was determined by RT-PCR and normalized to the expression level of *Actb*. Dampened M1 polarization in THP-1 *Gls* KO macrophages. Wild-type THP1 cells and *Gls* KO cells, generated by the Synthego Corporation, were subjected to differentiation and M. tuberculosis infection. mRNA expression of *IL1B* and *GLUT1* was analyzed by sm-RNA-FISH (F) as described in Fig. 1. FISH data are shown as means ±95% CI and represent three independent experiments. (G) Mitochondrial mass was evaluated using MitoView Fix 640 by flow cytometry and quantified (left: gating strategy; right: quantification). Data are shown as means ± S.D. from three independent experiments. Statistical significance at *, *P < *0.05, **, *P < *0.01, ***, *P < *0.001 and ****, *P < *0.0001 was based on two-tailed student’s *t* test.

To test findings from metabolomics studies, we investigated the kinetics of glutamine uptake in infected BMDMs by monitoring changes of glutamine levels in the culture supernatant. We found that a high rate of glutamine uptake at early phases of macrophage infection (up to 8 hpi) (Fig. 4A) coincided with M1-like polarization (refer to Fig. 1) and with increased mRNA molecules of the *Gls* gene (Fig. 4B), which encodes the mitochondrial glutaminase (GLS), a key enzyme in the glutaminolysis pathway. An increased dependence on glutamine by M. tuberculosis-infected human monocyte-derived macrophages and THP1 macrophages at 26 hpi has been reported (30), although the contribution of glutamine to M1 and/or M2 polarization is not clearly defined.

When we used GLS inhibitors BPTES and CB-839 (61–63) to validate the role of glutamine catabolism in M1-like polarization, we found that BPTES or CB-839 dampened M1-like polarization of BMDMS, as measured by decreased mRNA levels of M1 immunometabolic markers that included *Il1b*, *Nos2*, *Glut1*, *Pfkfb3*, and *Mct4* (Fig. 4C and Fig. S8). The dampening led to enhanced growth of M. tuberculosis in host cells (Fig. 4D). The inhibitory effects on macrophage polarization were alleviated by addition of dimethyl-α-ketoglutarate (DMKG), a membrane-permeable α-KG precursor (Fig. 4C and D and Fig. S8). Consistent with dampening of M1-like polarization by the inhibition of glutaminolysis, the GLS inhibitor BPTES also promoted M2 polarization, as evidenced by increased levels of *Arg1* mRNA that accompanied decreased *Il1b* mRNA levels in infected and inhibitor-treated BMDMs (Fig. 4E). This conclusion is further supported by the analysis of intracellular metabolites in BPTES-treated BMDMs at 8 hpi, which showed decreased levels of lactate as well as TCA intermediates and their derivatives that included succinate and itaconate (Table S2).

10.1128/mbio.01274-22.8 FIG S8 Diminished M1-like polarization by GLS inhibition with CB-839. BMDMs were infected with *M. tuberculosis*, and CB-839 was added to sets of cultures for GLS inhibition experiments at a final concentration of 10 μM. For rescue experiments, 1.5 mM of dimethyl α-ketoglutarate (DMKG) was added to sets of cultures treated with the inhibitor. mRNA molecules in infected BMDMs, with or without CB-839 and/or DMKG at 8 hpi, were detected and analyzed for *Il1b*, *Nos2*, and *Glut1* by sm-RNA-FISH as described in Fig. 1. sm-RNA-FISH data are shown as means ± 95% CI and represent three independent experiments. Statistical significance at *, *P* < 0.05, **, *P* < 0.01, ***, *P* < 0.001, and ****, *P* < 0.0001 was based on two-tailed student’s *t* test. Download FIG S8, TIF file, 0.8 MB. Copyright © 2022 Jiang et al. 2022 Jiang et al. https://creativecommons.org/licenses/by/4.0/ This content is distributed under the terms of the Creative Commons Attribution 4.0 International license.

10.1128/mbio.01274-22.10 TABLE S2 Decreased TCA cycle intermediates/derivatives in infected and BPTES-treated macrophages. Download Table S2, DOCX file, 0.01 MB. Copyright © 2022 Jiang et al. 2022 Jiang et al. https://creativecommons.org/licenses/by/4.0/ This content is distributed under the terms of the Creative Commons Attribution 4.0 International license.

As a test for the generality of our observations with murine BMDMs, we examined the expression of *IL1B* and *GLUT1* in M. tuberculosis-infected THP1 macrophages in which *GLS* was knocked out (KO). sm-RNA-FISH and mitochondrial mass staining with MitoView showed that at 8 hpi, mRNA levels of *IL1B* and *GLUT1* and the mitochondrial mass were lower in KO cells deficient in *GLS* (Fig. 4F and G). Since an increase in mitochondrial mass is associated with macrophage proinflammatory differentiation and response (64), the decreased mitochondrial mass suggests a compromised proinflammatory differentiation in *GLS* KO cells. These data demonstrate the importance of glutaminolysis in the proinflammatory response of human macrophages during M. tuberculosis infection.

## DISCUSSION

**FIG 5:**
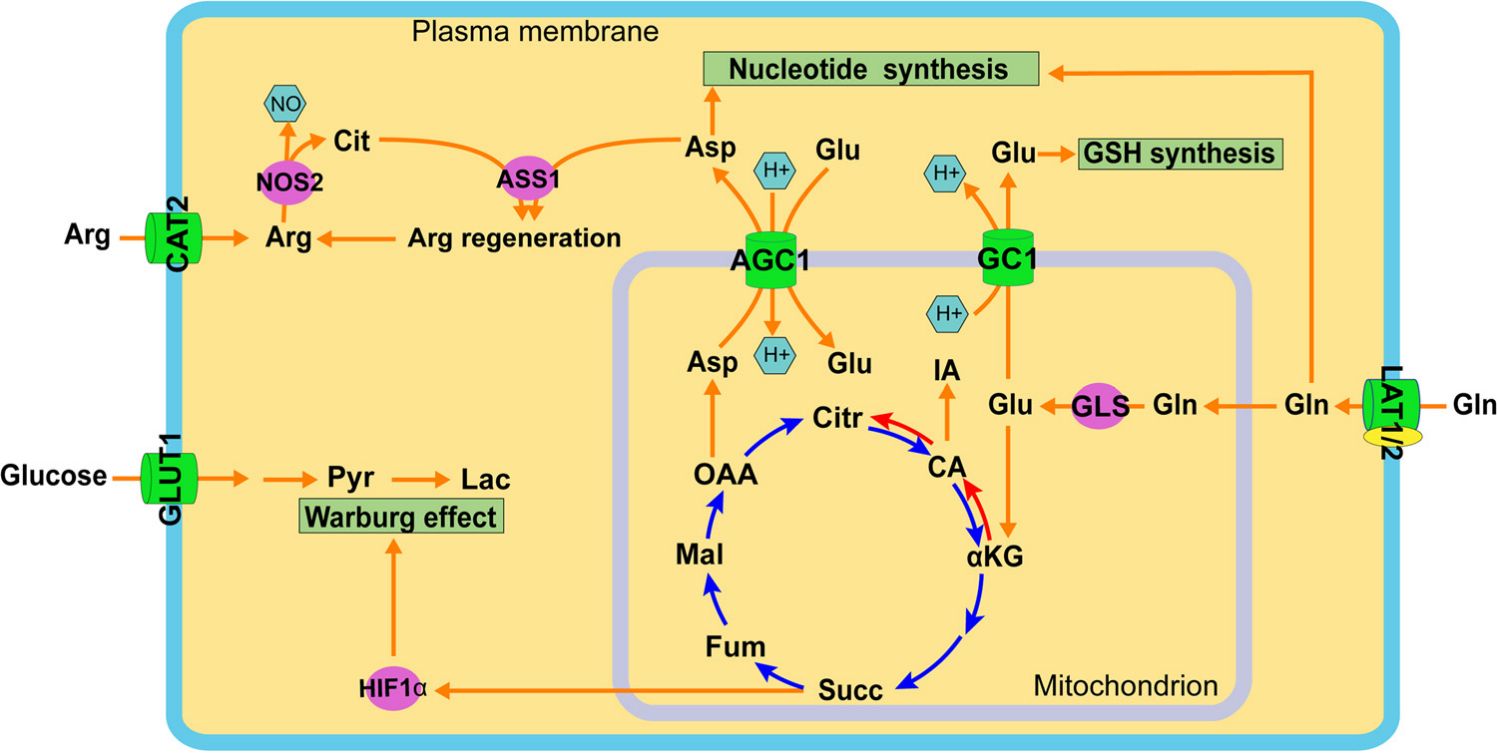
Coordination of glucose and glutamine catabolism during M1-like polarization. M1-like polarization is marked with increased catabolism of glucose and glutamine (Gln) in distinctive subcellular compartments but with coordinated functions. Increased glucose uptake, mediated by GLUT1, and its catabolism in the glycolysis pathway in the cytosol result in the production of lactate (Lac). Increased Gln uptake, probably mediated by neutral amino acid antiporters LAT1 and/or LAT2, and its direct metabolite glutamate (Glu) participate in multiple pathways of the metabolic remodeling program of M1-like macrophages. Glu, the product of the glutaminolysis pathway by glutaminase (GLS) in mitochondria, enters the TCA cycle for anaplerosis reactions. Glu replenishes the TCA cycle leading to the generation of itaconate (IA) and succinate, which promotes stabilization of HIF-1α and the subsequent Warburg effect. Gln also contributes both carbon and nitrogen to the formation of the nonessential amino acid aspartate (Asp), probably as a result of transamino reactions in mitochondria, which is then involved in biosynthetic pathways in the cytosol, including nucleotide synthesis and intracellular arginine regeneration by coupling with nitric oxide synthase 2 (NOS2)-derived citrulline (Cit) via argininosuccinate synthase 1 (ASS1) to sustain NO generation by NOS2. The export of mitochondrial Asp to the cytosol is mediated by the functional coupling between two mitochondrial carriers: the glutamate carrier 1 (GC1) exports the mitochondrial Glu deriving from GLS activity to cytosol, and cytosolic Glu then enters mitochondria by serving as a substrate of aspartate/glutamate carrier 1 (AGC1) for the net export of mitochondrial aspartate to the cytosol. Cytosolic Glu also participates in intracellular redox homeostasis involving the synthesis of glutathione (GSH). Abbreviations: CAT2, cationic amino acid transporter 2; αKG, α-ketoglutarate; Arg, arginine; CA, cis-aconitate; Citr, citrate; Fum, fumarate; Mal, malate; OAA, oxaloacetate; Pyr, pyruvate.

The findings described above indicate that glucose and glutamine play distinctive roles in the metabolic reprogramming during macrophage activation to the M1-like phenotype (Fig. 5). In addition to validating increased glycolytic flux from glucose catabolism to lactate during the M1-like polarization, our study identified glutamine catabolism as an integral component of the metabolic reprogramming of M1-like macrophages by serving as an important source of carbon and nitrogen.

A major role for glutamine catabolism during the M1-like polarization is to replenish the TCA cycle for the generation of intermediates (i) as signaling molecules, such as succinate, which is responsible for HIF-1 stabilization and the switch of glucose metabolism to glycolysis seen in LPS-activated M1 macrophages (14, 65–67) and (ii) as biosynthetic precursors for the synthesis of aspartate and itaconate. This anaplerotic function of glutamine carbons through the TCA cycle is fulfilled by simultaneous operation in the oxidative and reductive routes in M1-like macrophages, as confirmed by the formation of both M + 4 and M + 5 citrate from U^13^C glutamine. The relative contribution from each of the two routes to itaconate formation remains to be determined, as citrate with both M + 4 and M + 5 isotopologues leads to the formation of M + 4 itaconate (refer to Fig. 3A). Cytosolic IDH1 and mitochondrial IDH2 are known to catalyze the reductive carboxylation of glutamine-derived α-KG to isocitrate. In cancer cells, IDH1- or IDH2-mediated carboxylation of α-KG to citrate is crucial for cell growth and viability under hypoxia (54, 55). It is important to note, however, that the expression of both *Idh1* and *Idh2* is downregulated, at least at the mRNA level, in M1-like macrophages (supplemental and/or source data in references 31 and 68). Thus, additional work is needed to identify the enzyme responsible for the reductive glutamine metabolism during M1-like polarization.

Glutamine also serves as the carbon and nitrogen source for the formation of nonessential amino acids, such as aspartate and glutamate, which can potentially coordinate the metabolic reprogramming of M1-like macrophages in various subcellular compartments. For example, glutamate participates in the redox homeostasis of M1 macrophages through the synthesis of GSH directly by serving as a substrate and indirectly by coupling with antiporter xCT for the uptake of cystine, another precursor for GSH synthesis (44, 47). This scheme is supported by increased accumulation of glutamate, GSH, and GSSG in M. tuberculosis-induced M1-like macrophages (Fig. 2C and Fig. S3) and by the decreased GSH/GSSG in M. tuberculosis-infected THP1 *GLS* KO macrophages compared with WT control cells (Fig. S6B). Detection of GSH or GSSG by U^13^C glutamine tracing with silylation and GC-TOF/MS analysis is challenging due to their large molecular weight, as they must pass through the column under regular GC oven temperature settings. Additionally, aspartate can partake in arginine regeneration in the cytosol in conjunction with NOS2-derived citrulline via the argininosuccinate synthase 1 (ASS1)-mediated pathway for sustained NO production by NOS2 of M1 macrophages (Fig. 5) (23), especially when extracellular arginine levels are low (69). Intracellular arginine could not be measured reliably in the stable isotope experiments, probably due to its low level from the high expression of arginine-catabolizing enzymes such as NOS2 in M1 macrophages.

Aspartate, together with glutamine, participates in the synthesis of nucleotides required for M1 polarization, probably through the purine nucleotide cycle (57). Glutamine-derived aspartate could contribute to the purine nucleotide cycle, which helps maintain the balance of the glycolysis-mitochondrial redox interface by preventing cytoplasmic acidification of M1 macrophages (57). This hypothesis is supported by the recently identified role of FAMIN (fatty acid metabolism-immunity nexus) as a multifunctional purine nucleoside enzyme activity that enables the purine nucleotide cycle (57). Indeed, expression of genes encoding purine nucleotide cycle enzymes, including FAMIN, adenylosuccinate lyase, and AMP deaminase 3, is increased in M. tuberculosis-infected M1-like macrophages (supplemental and/or source data in references 31 and 68).

Given that glutamate and aspartate are produced predominately in mitochondria by GLS and transamination reactions, a metabolic coordination between mitochondria and the cytosol may constitute an important feature of the metabolic reprogramming of M1-like macrophages. Such coordination between the two compartments can be achieved by coupling two mitochondrial membrane transporters, the mitochondrial membrane glutamate carrier 1 (SLC25A22/GC1) and the aspartate-glutamate antiporter (AGC1/SLC25A12) (70) (Fig. 5). Gene expression for both transporters is increased in M. tuberculosis*-*infected M1-like macrophages (source data in reference 68). Indeed, the role of glutamine-derived aspartate in supporting cell growth and cellular redox homeostasis is supported by the observation that insufficient cytosolic aspartate delivery leads to cell death when TCA cycle carbon is reduced following glutamine withdrawal and/or glutaminase inhibition by the small-molecule inhibitor CB-839 (71). Collectively, the pleiotropic routes of glutamine catabolism linking metabolic pathways during M1-like polarization indicate a critical role for glutamine in the coordination of intracellular processes to meet the requirement of M1-like macrophages for bioenergetics and biosynthetic precursors (Fig. 5), as is needed for cell growth and proliferation (72, 73). Thus, our study identifies glutamine catabolism as an integral component of metabolic reprogramming in M1-like macrophages.

While glutamine anaplerosis generates succinate and itaconate in M1-like macrophages and inhibition of glutaminolysis by a GLS inhibitor decreases both metabolites and the M1-like polarization, the mechanism by which these metabolites contribute to the M. tuberculosis-induced M1-like polarization warrants further study. Itaconate produced by *Irg1* induction in activated macrophages correlates with the production of succinate via inhibition of succinate dehydrogenase (53, 74), which in turn leads to increased expression of HIF-1 and consequently increased glycolysis and M1 polarization (14). Increased *Irg1* expression in myeloid cells is required for M. tuberculosis control in both *in vitro* and *in vivo* settings (75, 76), indicating the critical role of itaconate for the antimicrobial response of host immunity against M. tuberculosis. This idea appears to be contradictory to the notion that itaconate is an immunosuppressive metabolite. The role of itaconate in the regulation of macrophage immunity is derived mainly from utilization of itaconate derivatives, including dimethyl itaconate (DI) and 4-octyl itaconate (4OI), which activate the anti-inflammatory response of macrophages via NRF2 (the master regulator of antioxidant response, also known as NFE2L2)-dependent and -independent pathways. Specifically, DI represses the expression of IL-6, IL-12, and pro-IL-1β induction in BMDMs by inhibiting IκBζ (human NFKB inhibitor zeta ortholog) protein induction through ATF3 (activating transcription factor 3) in an NRF2-independent manner (77). In contrast, 4OI decreases IL-1β mRNA, pro-IL-1β, and HIF-1α levels in activated BMDMs and human PBMCs, which NRF2 mediates by increasing the expression of downstream genes having antioxidant and anti-inflammatory capacities (78). However, recent studies show that neither DI nor 4OI converts to intracellular itaconate (79, 80) and that their anti-inflammatory role is associated mainly with the strong electrophilic stress response that involves GSH due to their strong electrophilic strength (77, 81). Thus, it is likely that itaconate with weak electrophilic strength may have a limited role in the anti-inflammatory response, at least at low concentration or at early stages of infection when the level of itaconate is low. Interestingly, during M. tuberculosis-induced M1-like polarization, mRNA levels for *Nfkbiz*, *Nfe2l2*, and *Atf3*, which encode IκBζ, NRF2, and ATF3, respectively, are induced in addition to *Irg1* (supplementary file in reference 31), indicating complex interactions among these regulators in M. tuberculosis-infected macrophages. Itaconate may promote the proinflammatory response at early stages of macrophage infection via succinate accumulation-mediated signaling and IκBζ upregulation, but then it may transition to function predominantly in an anti-inflammatory mode at high concentration through the induction of NRF2-dependent and -independent pathways as the infection progresses to the adaptation stage. It is important to note that the anti-inflammatory effects of itaconate are also mediated by other factors, such as production of reactive oxygen species, inhibition of glycolytic enzymes, and production of type I interferon (79, 82, 83). Thus, the complex and temporal correlations among these regulators with the immunometabolic state of infected macrophages and with M. tuberculosis growth dynamics require further clarification.

Our study has potential limitations. For example, the data were derived from a snapshot of the metabolic state of M1-like polarization during M. tuberculosis infection, and they do not necessarily represent the metabolic steady-state of M1-like macrophages. In addition, detection limitation of the isotope analytical systems, together with possible low concentrations of some metabolites, could be responsible for the absence of ^13^C enrichment for some metabolites. Nevertheless, the overall metabolomics data clearly demonstrate that glutamine contributes carbon and nitrogen to multiple cellular processes of the metabolic reprogramming that occurs during M. tuberculosis infection-induced M1-like polarization (Fig. 5). Since findings from cytokine quantitative trait locus analysis show that glutamine metabolism-related genes are associated with the cytokine response of human peripheral blood cells to M. tuberculosis infection (84), glutamine likely plays critical roles in host immunity against M. tuberculosis infection *in vivo*. The present study is a first step toward delineating the role of glutamine metabolic pathways at different pathophysiological states of TB.

It is now important to investigate how therapeutic targeting of host glutamine metabolism affects the metabolism and physiology of M. tuberculosis in infected macrophages, since glutamine also serves as a predominant source of nitrogen for synthesis of other amino acids during M. tuberculosis growth in human macrophages (60). A complex situation may arise in which supplementing glutamine to boost host immunity and better treatment outcome may also potentially stimulate pathogen growth. Thus, only a comprehensive understanding of glutamine metabolism in the context of host-pathogen interactions, in particular at different stages of the disease development, can guide the development of host-directed treatments to improve bacterial clearance and/or prevent the induction of immunopathology in tuberculosis.

## MATERIALS AND METHODS

### M. tuberculosis culture.

Cultures of green fluorescent protein (GFP)-M. tuberculosis H37Rv were grown under aerobic conditions at 37°C in Dubos Tween albumin (DTA) medium (Becton, Dickinson, Franklin Lakes, NJ) (85). Mid-log-phase cultures at an optical density at 580 nm (OD_580_) of 0.3 to 0.5 were used for infection.

### Ethics statement.

The use of mice for the generation of bone marrow macrophages in the study was approved by Institutional Animal Care and Use Committee (IACUC) in Rutgers (protocol no. PROTO999900960). The Public Health Research Institute (PHRI) animal facility in Rutgers is fully accredited by the American Association for Accreditation of Laboratory Animal Care and is operated in accordance with the Animal Welfare Act (AWA), the Public Health Service Research Extension Act (PHSREA), and all other policies administered by the USDA.

### Culture, differentiation, and infection of mouse BMDMs and THP1 cells.

Bone marrow cells isolated from femur and tibia of C57BL/6J mice were differentiated into BMDMs for 7 to 8 days by a standard procedure (86) in DMEM (Sigma, St. Louis, MO) supplemented with 10% fetal bovine serum (FBS; Thermo Fisher, Waltham, MA), 10% L929-cell-conditioning medium, and 1% penicillin/streptomycin (Corning, Glendale, AZ) (68). BMDMs were seeded into 6- and 24-well plates at a density of 1 × 10^6^ and 3 × 10^5^ cells per well, respectively. For sm-RNA-FISH, BMDMs were seeded onto gelatin-coated coverslips located at the bottom of 24-well plates. BMDMs were infected with M. tuberculosis at a multiplicity of infection (MOI) of 4 in culture medium for 4 h, and they were further cultured for up to 24 h after removal of extracellular bacteria. For GLS inhibition experiments, inhibitors CB-839 or BPTES (MedChemExpress LLC, Monmouth Junction, NJ) were added to sets of cultures 12 h prior to infection at a final concentration of 10 μM, according to literature (87, 88). For rescue experiments, dimethyl α-ketoglutarate (DMKG; Sigma, St. Louis, MO) was added to sets of cultures with either inhibitor at a final concentration 1.5 mM (89). After removal of extracellular bacteria at 4 hpi, fresh culture media containing the same concentration of either inhibitor or DMKG were added back to the corresponding wells for the duration of the experiments. BMDMs and supernatants were harvested at different times postinfection (p.i.) together with those from uninfected controls for various analyses. For M. tuberculosis CFU determination in macrophages, an MOI of 1 was used for infection, cells were lysed in 0.6% SDS solution at the indicated times, and CFU was determined by plating cell lysates on 7H11 agar plates following standard procedures (85).

THP1 cells (ATCC: TIB-202, Manassas, VA) were used to generate a pool of *Gls*-null cells. CRISPR/Cas9-mediated knockout (KO) cells were generated by the Synthego Corporation (Menlo Park, CA). *Gls* KO cells and their parental wild-type cells were cultured in RPMI cell culture medium (Sigma, St. Louis, MO) supplemented with 10% FBS (Thermo Fisher, Waltham, MA), differentiated by treatment with 100 μM phorbol 12-myristate 13-acetate (PMA; Sigma, St. Louis, MO) for 24 h, recovered for 24 h in fresh RPMI medium, and then infected as described for mouse BMDMs.

### sm-RNA-FISH.

sm-RNA-FISH probes were designed using Stellaris probe designer, an online tool (https://www.biosearchtech.com/products/rna-fish). About 50 3′-amino modified oligonucleotides for each mRNA transcript were obtained from LGC Biosearch (Petaluma, CA; the probe sequences will be provided upon request). Human transcript sequences were used for the experiments with THP-1 macrophages and mouse transcripts were used for the rest. Pooled oligonucleotides were coupled with tetramethylrhodamine (TMR), Texas Red, or Cy5 fluorophores and purified by high-performance liquid chromatography (HPLC), as detailed in reference 90. BMDMs at different times of infection were fixed by 10% formaldehyde and permeabilized in 70% ethanol. After equilibration in hybridization wash buffer (10% formamide [Thermo Fisher, Waltham, MA] in 2× saline-sodium citrate [SSC] buffer), cells were incubated overnight in hybridization buffer (10% formamide [Thermo Fisher, Waltham, MA], 10% dextran sulfate [Sigma, St. Louis, MO], 2 mM vanadyl-ribonucleoside complex [Sigma, St. Louis, MO], 0.02% RNase-free bovine serum albumin [Thermo Fisher, Waltham, MA], and 0.001% Escherichia coli tRNA [Sigma, St. Louis, MO]) containing labeled mRNA probes at 37°C. The pooled probe set for each target mRNA was used at 25 ng per hybridization reaction (50 μL). Following incubation, cells were washed in hybridization wash buffer followed by mounting with antifade buffer (Abcam, Cambridge, UK) before proceeding to imaging.

### Image acquisition and analysis.

Analysis of mRNA molecules was performed using a modified system developed by Raj et al. (90). Briefly, coverslips with stained cells were placed on an Axiovert 200 M inverted fluorescence microscope (Zeiss, Oberkochen, Germany) with a 63× oil-immersion objective (numerical aperture 1.4) and a 14 Prime sCMOS camera (both from Photometrics, Tucson, AZ), and the system was controlled by Metamorph image acquisition software (Molecular Devices, San Jose, CA). Images in differential interference contrast (DIC), GFP, and each of the other fluorescence channels corresponding to the fluorophores used in each probe set were obtained. Images from fluorescence channels consisted of 16 optical sections separated by 0.2 μm with an exposure time ranging from 1,500 to 2,000 ms. Image analyses were performed using modified image-processing programs in MATLAB R2021a (Natick, MA). Briefly, the boundaries of all cells in each field were first charted by manually tracing over their DIC image to avoid biases. Z-stacks of RNA images were then analyzed, and the number of spots corresponding to RNA molecules in each fluorescence channel was counted. The algorithm processes the images with a Laplacian filter and provides the user with a 3D plot for a region of the cells with spots, allowing users to provide a threshold to separate the noise from the signal. This threshold was then employed to calculate the number of spots within the boundaries of cells. Evidence for the accuracy of this algorithm in counting cellular RNA molecules has been published (33, 90).

### Profiling of widely targeted small metabolites by QTRAP 6500+ LC-MS/MS systems (ABSciex).

BMDMs obtained by standard procedure were seeded into 6-well plates (1 × 10^6^ cells per well) in DMEM with 10% FBS (Fisher Scientific, Waltham, MA), 4 mM glutamine, and 25 mM glucose but without pyruvate 1 day prior to infection. BMDMs were infected by M. tuberculosis in the above-described DMEM for 4 h and cultured for another 4 h after the removal of extracellular bacteria. Cells at 8 hpi and the corresponding uninfected control cells were harvested by centrifugation, and metabolites were extracted with 80% methanol with internal standards of small metabolites by multiple rounds of freeze-and-thaw cycles in liquid nitrogen and ultrasonic ice water bath, respectively. Metabolites in the extraction solvent were collected by centrifugation and dried under gentle nitrogen flow. Metabolites were reconstituted in 80% methanol and analyzed with ultraperformance liquid chromatography (UPLC) coupled with ABSciex 6500+ QTrap mass spectrometer (ABSciex, Framingham, MA). Briefly, metabolite separation was performed with a reverse phase ACE PFP-C_18_ column, and data were collected with a multiple reaction monitoring (MRM) mode, using MultiQuant software (Sciex, Framingham, MA) to enable identification and quantification of metabolites of interest. A pooled quality-control sample was injected six times and used to calculate the coefficients of variation (CV). Metabolites with a CV higher than 30% were excluded from the data analysis. Multivariate analysis of the data set was performed by SIMCA-p software (Sartorius, Goettingen Germany), and the partial least-squares-discriminant analysis (PLS-DA) model was used to demonstrate differences in the metabolic profiles between infected macrophages and uninfected controls. Analysis of pathway enrichment was carried out by Metaboanalyst (V5.0) (https://www.metaboanalyst.ca).

### Tracing metabolomics by U^13^C glutamine, U^15^N glutamine, and U^13^C glucose.

BMDMs were seeded into 6-well plates (1 × 10^6^ cells per well) in DMEM containing 10% dialyzed FBS (Thermo Scientific, Waltham, MA), 4 mM unlabeled glutamine, and 25 mM glucose 1 day before infection. One hour before the infection, the culture medium was replaced by DMEM containing 10% dialyzed FBS but without glutamine. BMDMs were infected by M. tuberculosis in DMEM supplemented with 10% dialyzed FBS and 4 mM 50% U^13^C glutamine (Cambridge Isotope Laboratories, Inc, Tewksbury, MA; 2 mM labeled and 2 mM unlabeled glutamine) for 4 h. After extracellular bacterial cells were removed, they were cultured for another 4 h in DMEM supplemented with 10% dialyzed FBS and 4 mM 50% U^13^C glutamine. For tracing experiments with U^15^N glutamine, 4 mM 50% U^15^N glutamine (2 mM labeled and 2 mM unlabeled glutamine in replacement of U^13^C glutamine) was applied to the cultures.

For tracing experiments with U^13^C glucose, BMDMs were seeded into 6-well plates (1 × 10^6^ per well) in DMEM with 10% dialyzed FBS (Thermo Scientific, Waltham, MA), 25 mM unlabeled glucose, and 4 mM glutamine 1 day before the infection. One hour before infection, the culture medium was replaced by DMEM with 10% dialyzed FBS (Thermo Scientific, Waltham, MA) but lacking glucose. BMDMs were infected by M. tuberculosis in DMEM supplemented with 10% dialyzed FBS and 25 mM 50% U^13^C glucose (Cambridge Isotope Laboratories, Inc, Tewksbury, MA; 12.5 mM labeled and 12.5 mM unlabeled glucose) for 4 h. After extracellular M. tuberculosis removal, cells were cultured for another 4 h in DMEM supplemented with 10% dialyzed FBS and 25 mM 50% U^13^C glucose.

Uninfected control sets under the respective culture conditions with 4 mM 50% U^13^C glutamine or U^15^N glutamine or 25 mM 50% U^13^C glucose were included for comparative analyses. Natural abundance subtraction samples were also prepared with unlabeled 4 mM glutamine or 25 mM glucose. Cells at 8 hpi and corresponding uninfected controls were detached and harvested by centrifugation. Metabolite extraction from cell pellets was performed with 80% methanol as described above. The extract was dried under gentle nitrogen flow and derivatized with a methyl-moximation (with 15 mg/mL methoxyamine in pyridine, 30°C for 90 min) and silylation {BSTFA [*N*,*O*-bis(trimethylsilyl)trifluoroacetamide] or MTBSTFA [*N*-methyl-*N*-tert-butyldimethylsilyltrifluoroacetamide], at 70°C for 60 min}. The samples were then analyzed with GC-TOF/MS (Waters, Milford, MA) with an electron impact mode and a DB-5MS column (Agilent, Santa Clara, CA) following our published protocol (50). The data were analyzed with Masslynx software (Waters, Milford, MA), and the enrichment calculation followed Jennings and Matthews (91).

The metabolites in the U^13^C glutamine experiment were derivatized with BSTFA, and those in the U^13^C glucose and U^15^N glutamine experiments were derivatized with MTBSTFA, after methoximation. The oven programs are slightly different for BSTFA and MTBSTFA derivatization. For BSFTA, the initial temperature was 60°C and kept for 1 min, followed by 10°C to 320°C, and kept at 320°C for 6 min. For MTBSTFA, the initial temperature was 80°C and kept for 1 min, followed by 15°C to 200°C, then 10°C to 320°C, and kept at 320°C for 6 min. With MTBSFTA, the enrichment of all the metabolites was calculated with their molecular ions with all the carbons or nitrogen(s). In the BSTFA derivatization, the enrichment of most of the metabolites was also calculated with the ions containing all the carbons in the molecules except aspartate and citric acid. The fragment used for aspartic acid enrichment calculation is 232, which contains 3 carbons (carbon 2 to 4). In the first round, U^13^C glutamine generates U^13^C labeled aspartic acid. Therefore, we considered M + 1, M + 2, and M + 3 of the fragment 232 as the enrichment of labeled aspartic acid M + 2, M + 3, and M + 4 in the experiment. For citric acid, we used the most abundant ion, 273, for the enrichment calculation, which contains 5 carbons (carbon 1 to 5). In the reduction reaction, U^13^C glutamine generates U^13^C α-ketoglutaric acid. The unlabeled carbon would add to the 6th position of citric acid. Therefore, the M + 5 of fragment 273 contained all the labeled carbons from glutamine in the reduction pathway. In the first round of the oxidation pathway, U^13^C glutamine generates U^13^C oxoacetate. The unlabeled carbon from acetyl coenzyme A (acetyl-CoA) adds to the 1, 2 (or 4, 5) position of the citric acid. The 6th position would be labeled carbon. The enrichment of M + 3 for the fragment 273 (position 1, 2, 3, 4, 5) would represent the enrichment of 4 carbons labeled citric acid (92).

### Glutamine measurement.

Culture supernatants from M. tuberculosis-infected BMDMs at 0, 4, 8, 16, 24, and 48 hpi were collected by filtering with 0.2 μM microcolumn by centrifugation. Glutamine in culture supernatant was determined using the glutamine/glutamate-glo assay kit (Promega, Madison, WI) and used for calculating the kinetics of glutamine uptake/utilization, following the manufacturer’s instructions.

### ELISA.

Culture supernatants from M. tuberculosis-infected BMDMs at 0, 4, 8, and 24 hpi were collected by filtering with 0.2 μM microcolumn by centrifugation and then subjected to measurement of IL-1β protein using the mouse IL-1β uncoated enzyme-linked immunosorbent assay (ELISA) kit (Invitrogen, Waltham, MA), following the manufacturer’s instructions.

### RNA extraction, reverse transcription, and quantitative PCR.

Total RNA extraction from BMDMs infected and treated with GLS inhibitor BPTES, as described above, was carried out using RNAzol reverse transcription (RT) column kit from Molecular Research Center (Cincinnati, OH) according to the manufacturer’s recommendations. RT was performed using Thermo Fischer SuperScript IV first strand synthesis system (Waltham, MA) based on the manufacturer’s recommendations. Briefly, primer annealing to template RNA using random hexamer was carried out at 65°C for 5 min, followed by chilling on ice. RT reactions with annealed RNA and RT reaction mix that contains SSIV buffer, dithiothreitol (DTT), RNase inhibitor, and Superscript IV reverse transcriptase were incubated at 23°C for 10 min followed by incubation at 52°C for 20 min; the RT reactions were terminated at 85°C for 10 min. Quantitative PCR was performed using Thermo Fisher Applied Biosystems Power SYBR green master mix (Waltham, MA). Gene-specific PCR primer pairs with ID numbers 6671509a1, 6680415a1, and 7106255a1 for murine *Actb*, *Il1b*, and *Arg1*, respectively, were obtained from PrimerBank-MGH-PGA (https://pga.mgh.harvard.edu) and synthesized by Integrated DNA Technologies (Coralville, IA). PCR was carried out in a Bio-Rad thermal cycler CFX real-time system (Hercules, CA) with the following parameters: 95°C for 10 min, followed by 40 cycles of 95°C for 15 s and 60°C for 1 min. Gene expression was normalized to expression levels of *Actb.*

### Measurement of TCA cycle intermediates/derivatives in the cell pellets by GC-MS.

BMDMs were infected by M. tuberculosis and/or treated with BPTES for 8 h. Cell harvesting and metabolite extraction were carried out following the widely targeted small metabolites screening protocol, and the derivatization was performed using the U^13^C glutamine protocol with BSTFA as the silylation reagent as described above. The samples were analyzed on a 5977 Agilent GC-MS (Agilent). Data analysis was performed with MassHunter software (Agilent). U^13^C succinate and U^13^C citrate were used as the internal standards.

### Mitochondrial mass quantification by flow cytometry.

Infected THP1 cells were stained with 100 nM MitoView FIX 640 (Biotium, Fremont, CA) at the indicated times. Cells were then fixed in 4% formaldehyde, collected, and analyzed on a BD FACS Celesta (Becton Dickinson, Franklin Lakes, NJ) to evaluate mitochondrial mass. Data were analyzed using FlowJo version 7.5.5 software (Tree Star Inc., Ashland, OR).

### Glutathione measurement.

Glutathione (GSH) and oxidized glutathione (GSSG) were quantified using the GSH/GSSG-Glo Assay (Promega, Madison, WI) following the manufacturer’s instructions. Briefly, ~6,000 wild-type (WT) or *Gls* KO THP-1 macrophages per well are seeded in 96-well plates and infected as described above. At the indicated times, cell culture supernatant was removed, and cells were lysed using total glutathione lysis reagent (for total GSH measurement) or oxidized glutathione lysis reagent (for GSSG measurement). Next, luciferin generation reagent was added, followed by a 30-min incubation at room temperature. Afterwards, luciferin detection reagent was added and incubated for 15 min at room temperature. Luminescence was then recorded on a Cytation 5 (Agilent, Santa Clara, CA). The ratio of GSH to GSSG was calculated.

### Statistics.

A 95% confidence interval (CI) and/or two-tailed student’s *t* test among groups were carried out for statistical significance analyses. Multivariant statistical analysis for the widely targeted metabolite data was performed with SIMCA-p software (Sartorius, Goettingen Germany). A PLS-DA model was performed with unit variant scaled data. The cutoff for the variable importance in the projection (VIP) value was set to 1. Pathway analysis was performed in Metaboanalyst (V5.0) (https://www.metaboanalyst.ca). Comparison of isotope distribution in metabolites between infected cells and uninfected controls was analyzed by two-tailed student’s *t* test using GraphPad Prism 8.0 (San Diego, CA).
