# Supplementary figures and images for: Glutamine Is Required for M1-like Polarization of Macrophages in Response to Mycobacterium tuberculosis Infection
Source: mBio. 2022 Jun 28;13(4):e01274-22. doi: 10.1128/mbio.01274-22 (PMC9426538; doi:10.1128/mbio.01274-22)

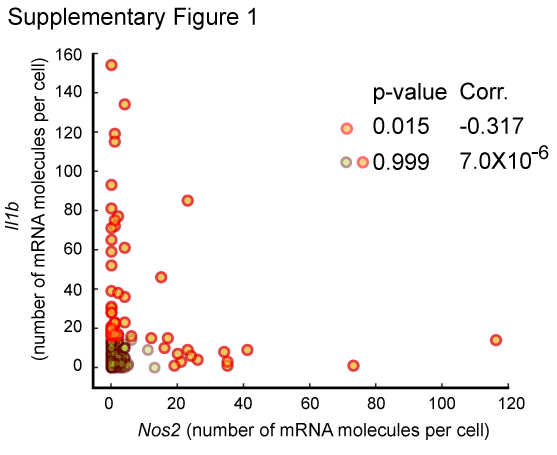

Supplement: FIG S1 [file mbio.01274-22-s0001.tif]

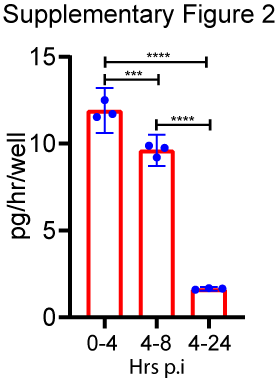

Supplement: FIG S2 [file mbio.01274-22-s0002.tif]

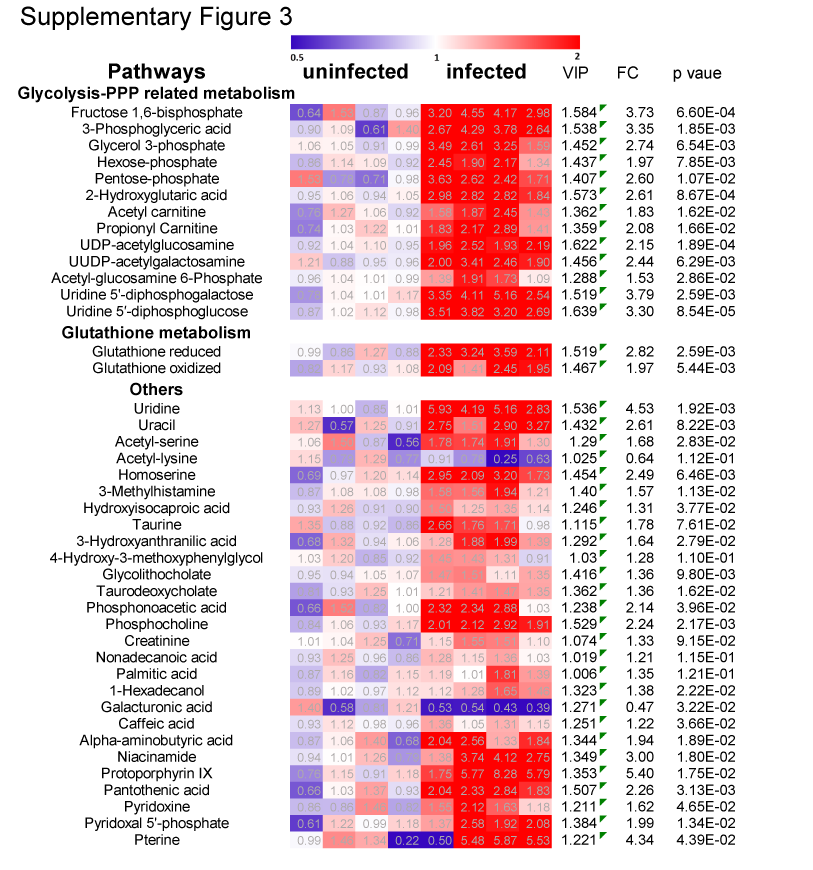

Supplement: FIG S3 [file mbio.01274-22-s0003.tif]

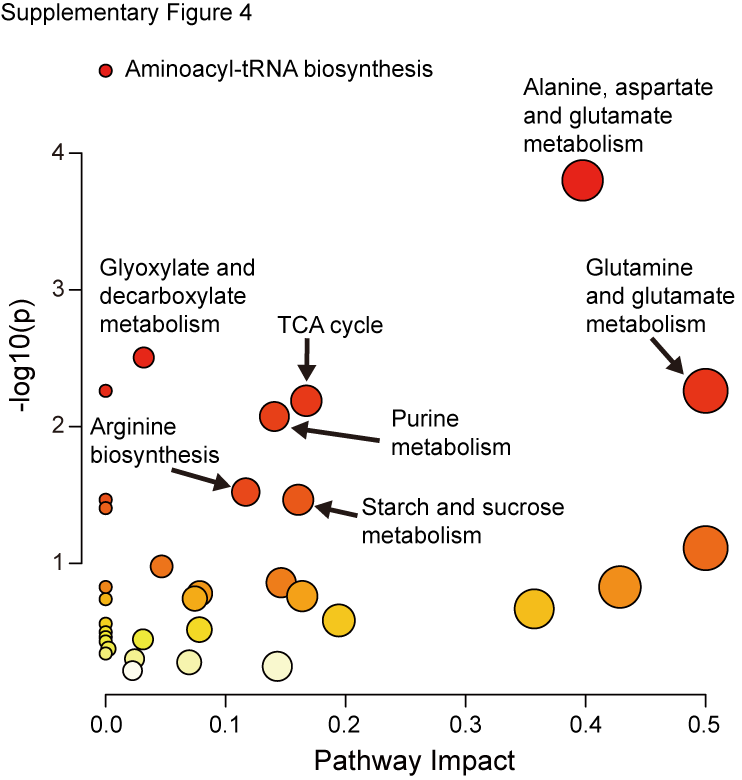

Supplement: FIG S4 [file mbio.01274-22-s0004.tif]

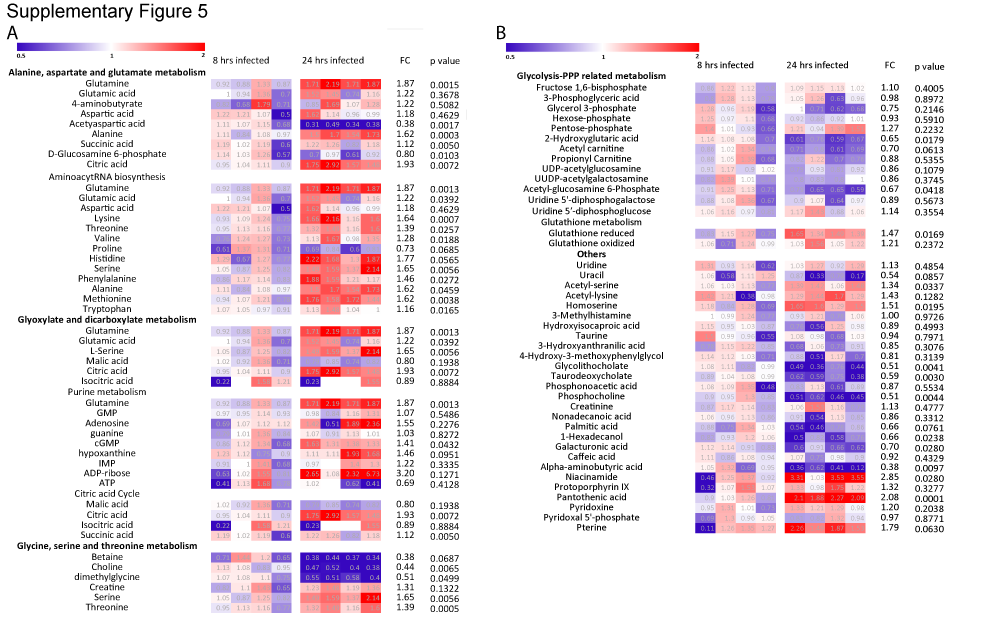

Supplement: FIG S5 [file mbio.01274-22-s0005.tif]

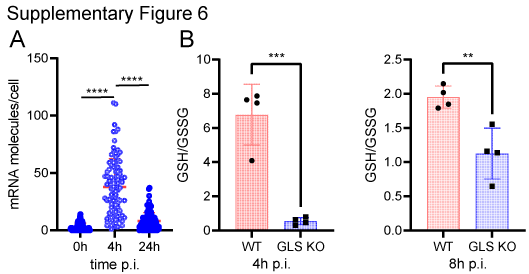

Supplement: FIG S6 [file mbio.01274-22-s0006.tif]

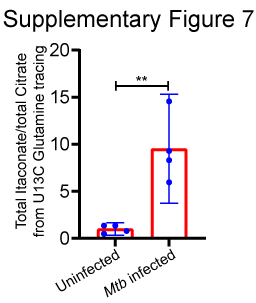

Supplement: FIG S7 [file mbio.01274-22-s0007.tif]

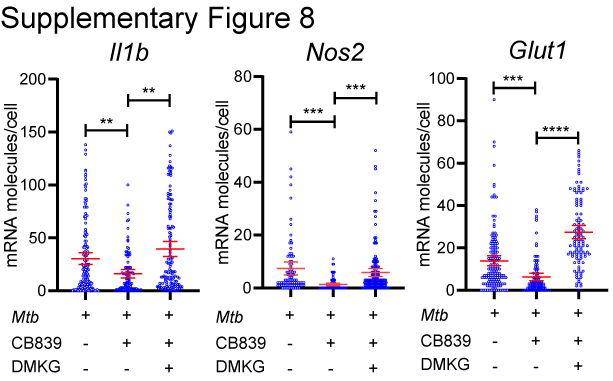

Supplement: FIG S8 [file mbio.01274-22-s0008.tif]
