# Supplementary material for: Glutamine Is Required for M1-like Polarization of Macrophages in Response to Mycobacterium tuberculosis Infection
Source: mBio. 2022 Jun 28;13(4):e01274-22. doi: 10.1128/mbio.01274-22 (PMC9426538; doi:10.1128/mbio.01274-22)
Supplement: TABLE S2 [file mbio.01274-22-s0010.docx]

**S. Table 2**. **Decreased TCA cycle intermediates/derivatives in infected and BPTES-treated macrophages.**

| **Metabolites** | **Average** | | **Fold Change** | **p value** |
| --- | --- | --- | --- | --- |
|  | **Infection** | **Infection+**  **BPTES** | **Infection+BPTES**  **vs. Infection** |  |
| Pyruvate | 90.30 | 84.72 | 0.94 | 2.73E-01 |
| Lactate | 124.17 | 38.39 | 0.31 | 1.40E-05 |
| Succinate | 1.53 | 0.32 | 0.21 | 1.26E-04 |
| Fumarate | 3.70 | 2.40 | 0.65 | 3.03E-03 |
| Malic acid | 3.79 | 0.16 | 0.04 | 2.54E-05 |
| a-KG | 0.16 | 0.06 | 0.40 | 1.23E-02 |
| Citrate | 0.75 | 0.12 | 0.16 | 1.14E-06 |
| Itaconate | 0.97 | 0.17 | 0.18 | 5.70E-05 |

BMDMs were infected by *M. tuberculosis* and/or treated with BPTES for 8 hrs. Cell harvesting and metabolite extraction were carried out following the widely targeted small-metabolites screening protocol, and the derivatization was performed using the U^13^C glutamine protocol with BSTFA as the silylation reagent as described in the material and methods section of the main text. The samples were analyzed with a 5977 Agilent GC-MS (Agilent). Data analysis was performed with MassHunter software (Agilent). U^13^C succinate and U^13^C citrate were used as internal standards. Data are derived from four replicates in each group. The values in the average columns are the average ratio of the intensity of each metabolite to that of the corresponding internal standard. P values are based on two-tailed Student’s T-test.
